# Impact of sleep disturbance on mental health in schoolchildren with atopic dermatitis: A population-based study

**DOI:** 10.1016/j.jdin.2025.08.013

**Published:** 2025-10-01

**Authors:** Kan-Hsuan Lin, Yu-Jun Chang, Tung-Ming Chang, Kuender D. Yang, Ching-Yuang Lin, Ko-Huang Lue, Hai-Lun Sun, Pei-Fen Liao, Jun-Kai Kao, Yi-Giien Tsai

**Affiliations:** a Department of Pediatrics, Changhua Christian Children’s Hospital, Changhua, Taiwan; b Institute of Biomedical Sciences, National Chung Hsing University, Taichung City, Taiwan; c Epidemiology and Biostatistics Center, Changhua Christian Hospital, Changhua, Taiwan; d Child Development Center, Changhua Christian Children’s Hospital, Changhua, Taiwan; e Department of Post-Baccalaureate Medicine, College of Medicine, National Chung Hsing University, Taichung, Taiwan; f Department of Pediatrics, Mackay Memorial Hospital, New Taipei City, Taiwan; g Institute of Clinical Medicine, National Yang Ming Chiao Tung University, Taipei, Taiwan; h Department of Microbiology & Immunology, National Defense Medical University, Taipei, Taiwan; i Department of Allergy, Immunology and Rheumatology, China Medical University Children's Hospital, Taichung, Taiwan; j Division of Pediatric Nephrology, Clinical Immunological Center, China Medical University Children's Hospital, Taichung, Taiwan; k School of Medicine, Chung Shan Medical University, Taichung, Taiwan; l Department of Pediatrics, Chung Shan Medical University Hospital, Taichung, Taiwan; m Doctoral Program in Tissue Engineering and Regenerative Medicine, National Chung Hsing University, Taichung City, Taiwan; n Frontier Molecular Medical Research Center in Children, Changhua Christian Children Hospital, Changhua County, Taiwan; o School of Medicine, Kaohsiung Medical University, Taichung, Taiwan

**Keywords:** allergic comorbidity, anxiety, atopic dermatitis, depression, mental health, sleep disturbance

## Abstract

**Objective:**

This Taiwanese population-based cross-sectional (CARE) study examined associations between sleep disturbances and mental health symptoms in adolescents with atopic dermatitis (AD).

**Method:**

A cross-sectional analysis was conducted among 14,675 adolescents (median age: 12.6 years; 49.7% male) using validated self-report questionnaires to assess anxiety (Screen for Child Anxiety Related Emotional Disorders) and depression (Patient Health Questionnaire-9). AD was identified via physician diagnosis and self-reported symptoms. Sleep quality, frequency of disturbances, and daytime sleepiness were evaluated using self-report. Logistic regression models adjusted for sex and psychosocial factors were used to assess associations with mental health outcomes.

**Results:**

Adolescents with AD (*n* = 3470) had higher anxiety (18.7% vs 16.0%) and depression (4.2% vs 2.4%) prevalence than health controls (*n* = 7865) (*P* < 0.001). Among adolescents with AD, weekly sleep disturbances, poor sleep quality, and daytime sleepiness were independently associated with 2.1, 1.8, and 1.6-fold higher risks of anxiety and 4.4, 2.7, and 2.1-fold higher risks of depression, after adjustment for sex and psychosocial factors. A dose-response relationship was observed between the sleep disturbance frequency and symptom severity.

**Conclusion:**

Sleep disturbances are strongly associated with increased anxiety and depression symptoms in adolescents with AD. Interventions focusing on sleep and psychosocial stress may improve mental health outcomes in this vulnerable population.

**Capsule Summary**

• Adolescents with atopic dermatitis have higher risks of anxiety and depression, associated with sleep disturbances in a dose-response manner, independent of sex and psychosocial stressors.
• This study highlights the possible need for routine mental health and sleep assessments in adolescents with atopic dermatitis.

## Introduction

Adolescence is a crucial developmental stage characterized by significant emotional, cognitive, and behavioral changes.^1^ Mental health disorders, particularly anxiety and depression, are increasingly prevalent during this stage, affecting approximately 14% of adolescents worldwide.^2^^,^^3^ Atopic dermatitis (AD), a chronic inflammatory skin condition affects approximately 15% to 20% of children and adolescents globally.^4^ AD significantly diminishes quality of life, often impairing emotional well-being, cognitive functioning, and peer relationships.^5^, ^6^, ^7^, ^8^ Adolescents with AD may experience unique psychosocial stressors, including social stigma, academic pressures, and sleep disturbances, which can significantly impact their mental health. Investigating these associations during adolescence is essential to inform early interventions and improve long-term mental health outcomes in this population.

Sleep disturbances are common in adolescents, especially those with chronic or neuropsychiatric conditions.^3^ A bidirectional, developmentally sensitive relationship exists between sleep disruption and internalizing symptoms; poor sleep may increase the risk of future mental health issues, while existing anxiety and depression can further impair sleep.^9^^,^^10^ Anxiety is frequently associated with shorter sleep duration, poor continuity, and increased sleep complaints.^11^^,^^12^ Up to 40% of individuals with insomnia also meet criteria for depression, underscoring the strong link between sleep and mood disorders.^13^

AD is a chronic inflammatory skin disease with a multifactorial pathogenesis involving immune dysregulation, epidermal barrier dysfunction, microbial imbalance, and genetic predisposition. A hallmark of AD is a Th2-skewed immune response, with IL-4 and IL-13 driving barrier impairment, pruritus, inflammation, and increased susceptibility to infection.^14^ Sleep disturbance, often triggered by nocturnal pruritus, is a common and distressing complication of AD.^15^ Patients with AD frequently experience insomnia symptoms such as difficulty falling asleep, frequent night awakenings, and nonrestorative sleep, leading to daytime fatigue and functional impairment. These sleep disruptions are strongly associated to elevated symptoms of anxiety and depression, and may negatively affect academic performance and social development.^4^^,^^16^ The mechanisms remain unclear but may involve inflammatory pathways, stress reactivity, and maladaptive coping strategies.^4^^,^^6^^,^^16^

Although AD has been associated with higher rates of anxiety and depression, recent Mendelian randomization studies suggest no direct causal link, implicating mediating factors such as sleep disturbance.^17^ Inconsistencies in previous findings may reflect methodological limitations, including small sample sizes and insufficient control for confounding variables.^18^, ^19^, ^20^ Anxiety and depression in adolescents with AD may result from the combined effects of physical discomfort, psychological distress, and social challenges.^7^^,^^21^

To address this gap, the study analyzed data from a large population-based cross-sectional to examine the associations between AD, multidimensional sleep disturbances, and symptoms of anxiety and depression in adolescents. Sleep was evaluated across 3 domains: disturbance frequency, subjective quality, and daytime sleepiness. Psychological symptoms were assessed using validated tools, with analyses adjusted for key sociodemographic and psychosocial factors. This study aims to clarify the role of sleep disturbance in the mental health burden of adolescents with AD and highlight the importance of integrated dermatologic and psychological evaluation and intervention.

## Materials and methods

### Data collection and study population

This study was part of the Changhua School Children’s Asthma Screening and Environmental Factors Survey and Health Promotion Project (CARE study), a large-scale, population-based cohort study in Taiwan initiated in 2017, prior to the COVID-19 pandemic.^22^, ^23^, ^24^ The study aimed to investigate respiratory and dermatologic conditions among adolescents and their associated environmental and psychosocial factors.

**Fig 1:**
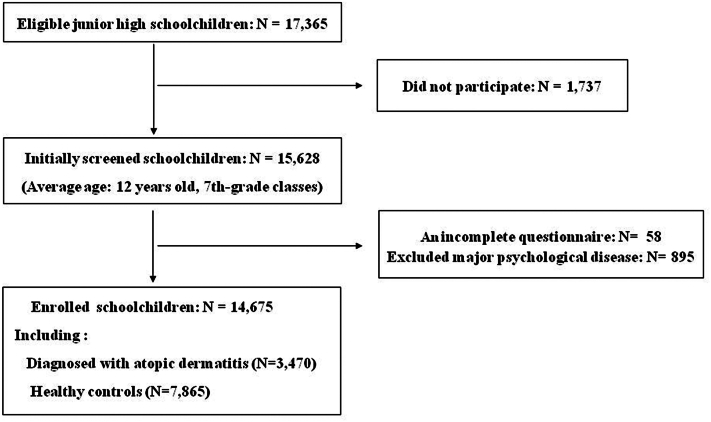
Schematic diagram of the participant enrollment process.

A total of 17,365 junior high school students were identified as eligible for participation. After accounting for 1737 refusals, 15,628 students (participation rate: 89.9%) were enrolled in the initial screening. The median age of the participants was 12 years (seventh grade). Pediatricians confirmed the diagnosis of AD based on a standardized clinical evaluation, which required: (1) a history of chronic or relapsing eczematous lesions persisting for at least 6 months, (2) characteristic morphology and distribution (eg, flexural involvement), and (3) the presence of pruritus. These criteria align with the Taiwan Guidelines for the Diagnosis and Management of Pediatric Atopic Dermatitis, as outlined in the consensus statement by the Taiwan Academy of Pediatric Allergy, Asthma, and Immunology.^25^ The excluded criteria included incomplete questionnaire data (*n* = 58) and a history of major psychiatric disorders (*n* = 895), resulting in a final analytic sample of 14,675 students (Fig 1). Among the participants, 3470 students had a confirmed diagnosis of AD and 7865 were classified as healthy controls. Healthy subjects were defined as students with no known chronic illnesses and no history of prior or current asthma, allergic rhinitis, or AD. Control participants were defined as students with no medically diagnosed chronic diseases and no self-reported history or current symptoms of asthma, allergic rhinitis, or eczema. These determinations were based on responses to the validated International Study of Asthma and Allergies in Childhood questionnaire^22^ (Fig 1).

Anxiety symptoms were assessed using the Screen for Child Anxiety Related Emotional Disorders (SCARED),^26^^,^^27^ and depressive symptoms were evaluated using the Patient Health Questionnaire-9 (PHQ-9).^28^ These self-administered instruments were completed in school settings under the supervision of trained school nurses to ensure accuracy and completeness. The study protocol was reviewed and approved by the Changhua Christian Hospital Institutional Review Board (IRB number 160207 and 160321). Written informed consent was obtained from all participants and their parents or legal guardians prior to data collection.

### Assessment of anxiety and depressive symptoms

Anxiety-related symptoms were assessed using the SCARED, a validated 41-item self-report instrument developed by Birmaher et al, and adapted for use in Chinese adolescent populations.^26^^,^^27^ The SCARED assesses a wide range of anxiety domains and has demonstrated robust psychometric properties in various settings. Each item is rated on a 3-point Likert scale (0 = not true or hardly ever true, 1 = somewhat true or sometimes true, 2 = very true or often true), giving a total score ranging from 0 to 82. A total score of ≥25 was used as the clinical cut-off point indicating clinically significant anxiety disorder. The instrument also includes validated subscale thresholds for specific anxiety domains: panic disorder/somatic symptoms (score ≥7), generalized anxiety disorder (score ≥9), separation anxiety disorder (score ≥5), social anxiety disorder (score ≥8), and significant school avoidance (score ≥3).

Depressive symptoms were evaluated using the PHQ-9, a nine-item self-report scale widely used to screen for depression in adolescents. Each item reflects one of the DSM-IV criteria for major depressive disorder and is rated from 0 (not at all) to 3 (nearly every day), resulting in total scores ranging from 0 to 27. The PHQ-9 has shown strong internal consistency, test-retest reliability, and validity compared to clinician-administered diagnostic interviews.^23^^,^^28^ A cutoff score ≥10 was used to identify participants with clinically significant depressive symptoms, which is consistent with standard practice in adolescents’ populations.

### Statistical analysis

Descriptive statistics were used to summarize the study population. Continuous variables are presented as median and interquartile ranges, while categorical variables are presented as absolute numbers and percentages. Group differences in demographic and clinical characteristics between adolescents with AD and those without were assessed using chi-square or Fisher’s exact tests for categorical variables, and Mann–Whitney *U* tests for continuous variables, given the nonparametric distribution of data. Within the AD group, Kruskal–Wallis tests assessed differences in SCARED and PHQ-9 scores by sleep disturbance frequency, with Bonferroni-adjusted post hoc tests as needed. Univariate linear regression identified sleep-related factors associated with emotional symptoms; significant variables were entered into multivariate models adjusting for key covariates. Analyses were performed using SPSS v22, with *P* < .05 indicating significance.

## Results

### Study population

A total of 14,675 adolescents (median age: 12.6 years; 49.7% male) were included (Table I). Clinically significant anxiety (SCARED ≥25) was present in 17.0%, and 2.5% had moderate-to-severe depressive symptoms (PHQ-9 ≥10). Sleep disturbances affected 31.5% overall, and 6.3% reported weekly episodes (Table I and Supplementary Table I, available via Mendeley at https://data.mendeley.com/datasets/bbjj7hpp64/1).

**Table I:** Demographic characteristics, sleep disturbances, and prevalence of childhood anxiety and depression in the study population (*n* = 14,675)

Total subjects (*n* = 14,675)	Mean	SD	Median	Q_1_	Q_3_
Sex, male (*n*, %)	7299 (49.7%)				
Age (y)	12.6	0.3	12.6	12.3	12.8
Height (cm)	139.2	38.7	153.1	146.2	159.0
Weight	64.3	39.8	48.6	41.0	63.0
BMI	20.1	4.2	19.1	17.1	22.2
Frequency of sleep disturbances (never)	10057 (68.5%)				
Frequency of sleep disturbances (<once a wk)	3695 (25.2%)				
Frequency of sleep disturbances (≥once a wk)	923 (6.3%)				
SCARED (total scores)	14.2	11.5	12.0	5.0	20.0
Panic disorder/somatization	2.7	3.3	2.0	0.0	4.0
Generalized anxiety disorder	3.4	3.4	3.0	0.0	5.0
Separation anxiety disorder	2.7	2.8	2.0	0.0	4.0
Social anxiety disorder	4.5	3.3	4.0	2.0	7.0
Significant school avoidance	0.8	1.2	0.0	0.0	1.0
Anxiety disorder					
(Total scores ≥25) (*n*, %)	2499 (17.0%)				
Panic/somatization					
(Items scores ≥7) (*n*, %)	1663 (11.3%)				
Generalized anxiety disorder					
(Items scores ≥9) (*n*, %)	1318 (9.0%)				
Separation anxiety disorder					
(Items scores ≥5) (*n*, %)	3376 (23.0%)				
Social anxiety disorder					
(Items scores ≥8) (*n*, %)	2431 (16.6%)				
Significant school avoidance					
(Items scores ≥3) (*n*, %)	1435 (9.8%)				
PHQ-9 (total scores)	2.0	2.9	1.0	0.0	3.0
Mild depression					
(Items scores >10-14) (*n*, %)	299 (2.0%)				
Moderate-severe depression					
(Items scores ≥15) (*n*, %)	81 (0.5%)				

*PHQ-9*, Depression Screen derived from Patient Health Questionnaire-9; *SCARED*, Scores for Child Anxiety-Related Disorders; *SD*, standard deviation.

Among adolescents with AD (*n* = 3470), the prevalence of sleep disturbances was higher (37.8%), with 7.8% reporting weekly disturbances and 10.9% reporting classroom sleepiness (Supplementary Table II, available via Mendeley at https://data.mendeley.com/datasets/bbjj7hpp64/1). These findings suggest a considerable emotional and sleep burden in this population, particularly among those with AD.

### Elevated anxiety and depression in adolescents with AD

Physician-diagnosed AD was identified in 23.6% of participants, and 19.5% reported recurrent itchy rash lasting at least 6 months. The discrepancy may reflect periods of remission in AD, during which pruritus subsides. Adolescents with visible eczema in typical locations had the highest anxiety (SCARED = 17.3) and depression (PHQ-9 = 3.0), followed by those with recent rashes in the past 12 months (SCARED = 17.0; PHQ-9 = 2.9). Those with AD diagnosis alone had lower scores (SCARED = 14.0; PHQ-9 = 2.0), indicating that active symptoms contribute more distress than diagnosis alone (Table II).

**Table II:** Prevalence of atopic dermatitis and associated with Scores for Child Anxiety-Related Disorders and Patient Health Questionnaire-9 scores in the adolescent study population (*n* = 14,675)

Indicator	*N*	%	SCARED (total scores)					PHQ-9 (total scores)				
			Mean	SD	Median	Q1	Q3	Mean	SD	Median	Q1	Q3
Recurrent itchy rash lasting at least 6 mo	2855	19.5	15.6	12.3	13	6	23	2.5	3.4	1	0	4
Recurrent itchy rash last 12 mo	1428	9.7	17.0	13.0	15	7	25	2.9	3.7	1	0	4
Eczema in typical locations	1073	7.3	17.3	12.9	15	7	25	3.0	3.7	2	0	5
Physician-diagnosed atopic dermatitis.	3470	23.6	14.0	11.4	12	5	20	2.0	2.8	1	0	3

*PHQ-9*, Patient Health Questionnaire-9; *SCARED*, Scores for Child Anxiety-Related Disorders; *SD*, standard deviation.

Compared to healthy controls (*n* = 7865), adolescents with AD (*n* = 3470) exhibited significantly higher anxiety (mean SCARED: 15.0 vs 13.6) and depression scores (mean PHQ-9: 2.3 vs 1.8; both *P* < .001). Clinically significant anxiety was more prevalent in the AD group (18.7% vs 16.0%; *P* < .001). Rates of mild (3.5% vs 2.0%) and moderate-to-severe depression (0.7% vs 0.4%) were also higher in the AD group (*P* < .05). (Table III). These findings highlight the elevated emotional burden associated with AD and support the need for routine mental health screening in dermatologic care.

**Table III:** Comparison of the Scores for Child Anxiety-Related Disorders and Patient Health Questionnaire-9 scores between adolescents with atopic dermatitis (*n* = 3470) and healthy controls (*n* = 7865)

	Diagnosed atopic dermatitis (*N* = 3470)					Health controls (*n* = 7865)					*P* value
	Mean	SD	Median	Q_1_	Q_3_	Mean	SD	Median	Q_1_	Q_3_	
Sex, male (*n*, %)	2762 (57.1%)					3588 (45.6%)					<.001
Age	12.6	0.3	12.6	12.3	12.9	12.6	0.3	12.6	12.3	12.8	.409
Height	139.7	38.0	153.2	146.5	159.0	138.6	39.2	153.0	146.0	159.0	.810
Weight	64.0	39.4	49.0	41.0	63.0	64.7	40.2	48.5	40.9	63.4	.754
BMI	20.1	4.2	19.2	17.1	22.5	20.0	4.3	19.1	17.1	22.2	.118
SCARED (total scores)	15.0	11.6	13.0	6.0	21.0	13.6	11.4	11.0	5.0	20.0	<.001
Panic disorder/somatization	3.0	3.4	2.0	1.0	4.0	2.5	3.2	2.0	0.0	4.0	<.001
Generalized anxiety disorder	3.7	3.5	3.0	1.0	6.0	3.3	3.4	2.0	0.0	5.0	<.001
Separation anxiety disorder	2.9	2.8	2.0	1.0	4.0	2.6	2.7	2.0	0.0	4.0	<.001
Social anxiety disorder	4.6	3.2	5.0	2.0	7.0	4.4	3.3	4.0	2.0	7.0	.002
Significant school avoidance	0.9	1.2	0.0	0.0	1.0	0.8	1.2	0.0	0.0	1.0	<.001
Anxiety disorder											
(Total scores ≥25) (*n*, %)	649 (18.7%)					1261 (16.0%)					<.001
Panic/somatization											
(Items scores ≥7) (*n*, %)	449 (12.9%)					795 (10.1%)					<.001
Generalized anxiety disorder											
(Items scores ≥9) (*n*, %)	351 (10.1%)					672 (8.5%)					.007
Separation anxiety disorder											
(Items scores ≥5) (*n*, %)	834 (24.0%)					1729 (22.0%)					.016
Social anxiety disorder											
(Items scores ≥8) (*n*, %)	593 (17.1%)					1293 (16.4%)					.392
Significant school avoidance											
(Items scores ≥3) (*n*, %)	373 (10.7%)					720 (9.2%)					.008
PHQ-9 (total scores)	2.3	3.2	1.0	0.0	4.0	1.8	2.7	1.0	0.0	3.0	<.001
Mild depression											
(Items scores >10-14) (*n*, %)	120 (3.5%)					160 (2.0%)					<.001
Moderate-severe depression											
(Items scores ≥15) (*n*, %)	26 (0.7%)					34 (0.4%)					.032

*P* value by Mann-Whitney *U* test.

*BMI*, Body mass index; *PHQ-9*, Patient Health Questionnaire-9; *SCARED*, Scores for Child Anxiety-Related Disorders; *SD*, standard deviation.

Female adolescents with AD showed significantly higher anxiety scores than males across all domains (mean SCARED: 16.4 vs 13.7; *P* < .001). Depression scores were slightly higher in females (PHQ-9: 2.4 vs 2.2; *P* < .05), though depressive symptom prevalence was similar. These findings indicate greater anxiety vulnerability in females with AD (Supplementary Table III, available via Mendeley at https://data.mendeley.com/datasets/bbjj7hpp64/1).

### Sleep disturbances and emotional symptoms in adolescents with AD

Generalized linear models revealed that high anxiety (SCARED) and depressive (PHQ-9) scores were independently associated with female sex, household smoking, prolonged screen time, academic, peer, and family pressures, as well as frequent sleep disturbances (*P* < .01). In the full adolescent population (*n* = 14,675), AD was an independent predictor of anxiety (*P* < .05), but not depression (Table IV).

**Table IV:** Generalized linear model estimates for the total scores of Scores for Child Anxiety-Related Disorders and Patient Health Questionnaire-9 in the adolescent study population (*n* = 14,675)

Parameter	SCARED (total scores)				PHQ-9 (total scores)			
	Estimate	SE	95% CI	*P* value	Estimate	SE	95% CI	*P* value
Sex								
Female	2.10	0.19	1.74-2.47	<.001	0.13	0.05	0.03-0.22	.007
Male	0.00				0.00			
BMI	−0.01	0.02	−0.05-0.03	.634	0.02	0.01	0.00-0.03	.005
Parental education level								
Middle school (or below)	2.04	0.31	1.43-2.66	<.001	0.13	0.08	−0.02-0.29	.090
High school/vocational	0.56	0.20	0.16-0.95	.006	−0.01	0.05	−0.10-0.09	.920
College (or above)	0.00				0.00			
Household smokers	0.57	0.11	0.35-0.78	<.001	0.14	0.03	0.09-0.19	<.001
Frequency of vigorous exercise								
Rarely	0.78	0.25	0.28-1.28	.002	0.00	0.07	−0.13-0.13	.968
1-2 times a wk	0.44	0.27	−0.09-0.96	.105	−0.01	0.05	−0.11-0.10	.922
≥3 times a wk	0.00				0.00			
Daily television viewing hours								
≥3 h	0.90	0.27	0.38-1.42	.001	0.20	0.07	0.07-0.33	.002
<3 h	0.00				0.00			
Sleep disturbance frequency								
≥3 times a wk	8.79	0.81	7.19-10.38	<.001	4.46	0.20	4.06-4.85	<.001
1-2 times a wk	6.40	0.43	5.56-7.24	<.001	2.57	0.11	2.36-2.78	<.001
<Once a wk	2.86	0.21	2.44-3.28	<.001	1.11	0.05	1.00-1.21	<.001
Never	0.00				0.00			
Academic pressure								
Yes	4.64	0.18	4.28-5.00	<.001	1.05	0.05	0.96-1.14	<.001
No	0.00				0.00			
Peer influence pressure								
Yes	4.66	0.25	4.17-5.15	<.001	0.92	0.06	0.80-1.04	<.001
No	0.00				0.00			
Family influence pressure								
Yes	4.03	0.28	3.49-4.58	<.001	1.35	0.07	1.21-1.49	<.001
No	0.00				0.00			
Diagnosed atopic dermatitis								
Yes	0.52	0.22	0.09-0.95	.017	0.10	0.05	−0.01-0.20	.074
No	0.00				0.00			

Adjusted for sex, BMI, parental education level, household smokers, frequent vigorous exercise, daily television viewing hours, frequency of sleep disturbances, academic pressure, peer influence pressure, family influence pressure and diagnosed atopic dermatitis.

*BMI*, Body mass index; *CI*, confidence interval; *PHQ-9*, Patient Health Questionnaire-9; *SCARED*, Scores for Child Anxiety-Related Disorders; *SD*, standard deviation.

In the AD subgroup, logistic regression identified clinically significant anxiety was associated with frequent sleep disturbances (odds ratio [OR], 2.08), poor sleep quality (OR, 1.80), and daytime sleepiness (OR, 1.60; all *P* < .01). Similar associations were observed for depressive symptoms: sleep disturbances (OR, 4.38), poor sleep quality (OR, 2.68), and daytime sleepiness (OR, 2.09; all *P* < .05) (Table V).

**Table V:** Logistic regression analysis of sleep disturbances in relation to anxiety disorder and depression among adolescents with atopic dermatitis (*n* = 3470)

Parameter	Anxiety disorder (SCARED ≥ 25)			Depression (PHQ-9 > 10)		
	Odds ratio	95% CI	*P* value	Odds ratio	95% CI	*P* value
Parents' education level						
Middle school (or below)	1.00			1.00		
High school/vocational	0.90	0.62-1.31	.587	0.98	0.43 -2.27	.970
College (or above)	0.74	0.50 -1.08	.119	1.06	0.46 -2.47	.888
Household smokers	1.16	0.96 -1.40	.127	0.97	0.64-1.45	.866
Frequency of vigorous exercise						
≥3 times a wk	1.00			1.00		
1-2 times a wk	1.31	0.99-1.73	.059	0.89	0.48-1.64	.713
Rarely	1.46	1.12-1.89	.004	1.48	0.87-2.52	.152
Daily television viewing hours						
≥3	1.09	0.84-1.42	.494	1.71	1.05-2.78	.030
<3	1.00			1.00		
Daily sleep hours						
>7 h	1.00			1.00		
6-7 h	1.13	0.93-1.38	.211	1.25	0.81-1.93	.311
<6 h	1.37	0.97-1.95	.078	1.99	1.07-3.71	.029
Sleep quality in the last month						
Very good	1.00			1.00		
Quite good	1.16	0.89-1.51	.275	1.40	0.67-2.92	.370
Poor	1.80	1.29-2.51	.001	2.68	1.20-5.98	.016
Sleep onset time						
≤30 min	1.00			1.00		
31-60 min	0.79	0.58-1.07	.122	0.63	0.35-1.14	.129
≥61 min	1.29	0.84-1.97	.240	0.84	0.33-2.14	.720
Frequency of sleep disturbances						
Never	1.00			1.00		
<Once a wk	1.21	0.98-1.49	.079	1.73	1.08-2.77	.022
≥Once a wk	2.08	1.50-2.90	<.001	4.38	2.46-7.78	<.001
Daytime sleepiness in class						
Never	1.00			1.00		
<Once a wk	1.11	0.89-1.38	.350	0.82	0.50-1.35	.440
≥Once a wk	1.60	1.21-2.10	.001	2.09	1.29-3.37	.003
Academic pressure						
Yes	2.39	1.97-2.91	<.001	1.98	1.27-3.07	.002
No	1.00			1.00		
Peer influence pressure						
Yes	2.04	1.65-2.52	<.001	1.75	1.14-2.67	.010
No	1.00			1.00		
Family influence pressure						
Yes	1.64	1.30-2.09	<.001	2.79	1.82-4.27	<0.001
No	1.00			1.00		

Adjusted for parental education level, household smokers, frequent of vigorous exercise, daily television viewing hours, sleep disturbance frequency, academic pressure, peer influence pressure, and family influence pressure.

*CI*, Confidence interval; *PHQ-9*, Patient Health Questionnaire-9; *SCARED*, Scores for Child Anxiety-Related Disorders.

### Incremental impact of sleep disturbances on emotional symptoms

Across the full population and the AD subgroup, poor sleep parameters, including shorter sleep duration, lower subjective sleep quality, more frequent sleep disturbances, and greater daytime sleepiness, were consistently associated with high anxiety and depression scores (*P* < .001; Supplementary Tables I and II, available via Mendeley at https://data.mendeley.com/datasets/bbjj7hpp64/1). We conducted additional generalized linear model analyses to compare the effects of sleep disturbance domains on anxiety and depression symptoms in adolescents with AD. Poor sleep quality showed the strongest associations with both SCARED (β = 4.81) and PHQ-9 (β = 2.03) scores. This was followed by frequent night awakenings, daytime sleepiness, and short sleep duration, all significantly associated with higher anxiety and depression scores (*P* < .01) (Table VI). As shown in Supplementary Fig 1, available via Mendeley at https://data.mendeley.com/datasets/bbjj7hpp64/1, both SCARED and PHQ-9 scores increased in a dose-dependent manner with the frequency of sleep disturbances (*P* < .05). These findings emphasize the critical role of sleep hygiene in adolescent mental health, particularly among those with AD.

**Table VI:** Associations between sleep disturbance domains and total Scores for Child Anxiety-Related Disorders and Patient Health Questionnaire-9 scores among adolescents with atopic dermatitis (*n* = 3470)

Parameter	SCARED (total scores)			PHQ-9 (total scores)		
	Estimate	95% CI	*P* value	Estimate	95% CI	*P* value
Daily sleep hours						
≤7 h	1.68	0.92-2.43	<.001	0.70	0.49-0.90	<.001
>7 h	0.00			0.00		
Sleep quality						
Poor	4.81	3.82-5.80	<.001	2.03	1.76-2.30	<.001
Good	0.00			0.00		
Sleep disturbances						
Yes	3.29	2.52-4.06	<.001	1.53	1.32-1.73	<.001
No	0.00			0.00		
Daytime sleepiness in class						
Yes	1.89	1.11-2.68	<.001	0.84	0.62-1.05	<.001
No	0.00			0.00		

Generalized linear models for total SCARED and PHQ-9 scores were adjusted for sex, BMI, parental education level, household smokers, frequent vigorous exercise, daily television viewing hours, academic pressure, peer influence, and family influence.

*CI*, Confidence interval; *PHQ-9*, Patient Health Questionnaire-9; *SCARED*, Scores for Child Anxiety-Related Disorders.

## Discussion

Adequate sleep is essential for emotional, cognitive, and behavioral functioning in school-aged adolescents, a population particularly vulnerable to internalizing disorders and poor academic performance when sleep is disrupted.^12^ In this population-based study of 14,675 adolescents, sleep disturbances were significantly associated with increased anxiety and depression among those with AD. Adolescents with AD reported more frequent sleep problems and higher rates of anxiety and depression symptoms compared to controls. Sleep disturbances are commonly reported in individuals with AD, with prevalence estimates ranging from 47% to 80% in children^18^^,^^29^ and 33% to 90% in adults.^30^^,^^31^ In this study, 37.8% of adolescents with physician-diagnosed AD reported sleep disturbances; 7.8% experienced them at least weekly, and 10.9% reported daytime sleepiness. Poor subjective sleep quality showed the strongest association with anxiety and depression, suggesting it may be a key indicator of sleep-related psychological distress in adolescents with AD. Frequent night awakenings were also closely linked to anxiety, while daytime sleepiness had moderate associations, reflecting the impact of poor sleep on daytime functioning and mood. Short sleep duration had the weakest but still significant associations, indicating that sleep quality may be more important than duration in relation to mental health outcomes.

Nocturnal pruritus and fragmented sleep may affect emotional regulation and cognitive function, increasing the risk of anxiety, depression, and other internalizing symptoms.^4^^,^^30^^,^^32^ In this population-based study, adolescents with AD reported poorer sleep quality, more frequent disturbances, and greater daytime fatigue compared to their peers without AD. Sleep problems were independently associated with elevated anxiety and depression scores, measured by the SCARED and PHQ-9 instruments. A clear dose-response relationship was observed between the frequency of sleep disturbances and the severity of emotional symptoms. These results suggest that cumulative sleep disturbances significantly contribute to the severity of anxiety and depression in adolescents with AD. These findings reinforce and extend the previous literature by quantifying the burden of sleep disturbances on emotional symptoms in adolescents with AD during a key developmental stage.^33^

Patients with AD may present with mild, moderate, or severe disease, often characterized by persistent pruritus that can disrupt sleep and contribute to psychological issues such as anxiety and depression, significantly affecting quality of life.^7^^,^^18^ Our results further indicate adolescents with recent disease flares or eczema involvement in typical anatomical location reported higher levels of anxiety and depression than those with a diagnosis alone, reflecting the psychosocial burden of visible or symptomatic skin disease. These results align with prior evidence showing that the severity, chronicity, and visibility of AD are strongly associated with emotional distress, diminished self-image, and impaired social functioning.^8^^,^^34^

Meta-analytic evidence indicates that AD patients have a significantly elevated risk of anxiety (OR = 1.62).^7^^,^^35^ In our population-based population of adolescent, physician-diagnosed AD was independently associated with anxiety, but not depression, after adjustment for sex and psychosocial stressors. This suggests a stronger link between AD and anxiety in adolescents compared to adults, which could reflect the earlier onset of anxiety disorders, which often precede depressive symptoms in youth.^36^^,^^37^ Anxiety frequently co-occurs with sleep disturbances in AD, including insomnia characterized by poor sleep quality, frequent awakenings, and reduced duration.^16^ In our sample, nearly 20% of adolescents with AD met the criteria for clinically significant anxiety, and those with frequent sleep disturbances had a 2.1-fold increased risk of anxiety symptoms.

Depression is also strongly associated with sleep disturbances, and insomnia affects up to 80% of individuals experiencing depressive symptoms. Furthermore, approximately 40% of people with insomnia meet the criteria for clinical depression, supporting a bidirectional relationship between the 2 conditions.^12^^,^^13^^,^^38^ Meta-analytic data further indicate that individuals with AD are at increased risk for depression (OR = 1.60) and suicidal ideation (OR = 1.44).^5^^,^^7^^,^^35^^,^^39^^,^^40^ In our study, school-aged adolescents with AD reported higher rates of depressive symptoms, with those frequent sleep disturbances had a 4.4-fold increased risk of clinically significant depression, highlighting sleep disruption as a key contributor to emotional burden in this population.

Female adolescents in our study reported higher levels of anxiety and slightly greater depressive symptoms, consistent with epidemiological data indicating increased vulnerability to anxiety in this group. These differences may be influenced by hormonal factors—such as fluctuations in estrogen during puberty—along with developmental aspects, including heightened emotional reactivity and rumination.^41^ Cultural and social influences, such as gendered expectations, body image concerns, and increased sensitivity to peer evaluation, may further contribute to the mental health burden in female adolescents with AD.^9^^,^^41^^,^^42^

Participants with major psychological disorders were excluded from the analysis to minimize confounding from pre-existing mental health conditions (Fig 1). However, we acknowledge the potential for residual confounding from unmeasured factors, such as undiagnosed psychiatric disorders or a family history of mental illness. Unlike earlier research limited by small or selective samples and inadequate psychosocial data, this study used a large, population-based cross-sectional design and validated instruments to provide a more comprehensive assessment of the emotional burden associated with AD. Although the cross-sectional design limits causal inference and reliance on self-report may introduce bias, the study’s strengths—large sample size, clinical diagnoses, and adjustment for confounders—enhance the validity and generalizability of the findings.

Our results identify sleep disturbance as a modifiable factor contributing to emotional distress in adolescents with AD. Routine screening for sleep and mental health symptoms in dermatologic care may improve early identification and intervention. Behavioral approaches to improving sleep may offer a practical and effective strategy to reduce emotional symptoms and enhance quality of life. Future longitudinal studies are warranted to examine causal pathways and evaluate whether improving sleep mitigates psychiatric symptoms in this vulnerable population.

## Conflicts of interest

None disclosed.
